# Value of 3.0 T diffusion-weighted imaging in discriminating thecoma and fibrothecoma from other adnexal solid masses

**DOI:** 10.1186/1757-2215-6-58

**Published:** 2013-08-21

**Authors:** He Zhang, Guo-Fu Zhang, Tian-Ping Wang, Hao Zhang

**Affiliations:** 1 Department of Radiology,Obstetrics and Gynecology Hospital, Fudan University, No. 419, Fang Xie Road, Shanghai 200011, China; 2 Department of Radiology, Shanghai First People’s Hospital, Medical College, Shanghai Jiaotong University, Shanghai 200080, China

**Keywords:** Thecoma, Ovarian solid mass, Magnetic resonance imaging, Diffusion-weighted imaging

## Abstract

**Background:**

To investigate the value of diffusion-weighted imaging (DWI) at 3.0 T (3T), and especially the apparent diffusion coefficient (ADC), in discriminating ovarian thecoma from other adnexal solid masses.

**Methods:**

Eighteen thecomas or fibrothecomas, 14 ligamentous leiomyomas, and 24 other ovarian solid tumors underwent prospective DWI magnetic resonance imaging (MRI) in addition to routine MRI on a 3T MRI machine. The baseline characteristics, components, and conventional MRI and DWI-MRI signals for the thecomas were recorded. The ADC values (ADCs) were measured for each group and compared.

**Results:**

The thecomas often appeared as homogeneous isointensity (17/18) on T1-weighted images (T_1_WI; 11/18) or T_2_WI (11/18) on DWI-MRI, with minor (9/18) or mild (6/18) enhancement. The mean ADC value for thecoma (1.20 ± 0.45 × 10^−3^ mm^2^/s) was almost equal to that of the other solid ovarian masses (1.26 ± 0.51 × 10^−3^ mm^2^/s), but lower than that for leiomyoma (1.48 ± 0.42 × 10^−3^ mm^2^/s), although not significantly so. There was a significant difference (*p* = 0.043) in the ADCs of the benign ovarian solid masses (1.16 ± 0.47 × 10^−3^ mm^2^/s) and leiomyomas (1.48 ± 0.42 × 10^−3^ mm^2^/s).

**Conclusions:**

There is no significant difference in ADC between thecoma and other adnexal solid masses, but the ADCs of thecomas are lower than those of leiomyomas.

## Background

Thecomas are rare, solid sex-cord stromal ovarian tumors, and account for approximately 0.5%–1% of primary ovarian lesions [1]. Although thecomas are often mixed with fiber components (then called “fibrothecomas”), thecomas and fibrothecomas are now considered to originate from the ovarian medulla, with a different etiology from fibromas, which originate from the cortex [2]. Because their prevalence is so low, the imaging features of thecomas are still not fully known [3,4]. Thecoma and fibrothecoma are the most common solid primary ovarian tumors and are frequently misdiagnosed as uterine fibroids [5]. Therefore, a better understanding of the imaging features of thecoma is of paramount importance for radiologists to ensure a correct preoperative diagnosis of this disease. Magnetic resonance imaging (MRI) is a second-line imaging modality with superb soft-tissue resolution that is widely used in the clinical context to examine any adnexal masses that are indeterminate on either palpation or ultrasonography(US), with promising results [6-9]. In recent studies, perfusion-MRI and diffusion-weighted imaging (DWI)-MRI have been used to distinguish malignant ovarian tumors from benign conditions [10,11]. However, to the best of our knowledge, 3.0 Tesla (3T) DWI-MRI evaluation of thecomas has not yet been reported. Therefore, by evaluating thecomas on a 3T MRI unit in this study, we aimed to: (1) describe the DWI characteristics of thecomas and record the apparent diffusion coefficient (ADC) for each lesion; (2) compare these features with those of adnexal leiomyomas and other primary and secondary solid ovarian tumors to determine whether DWI-MRI is useful in the differentiation of thecomas from other solid adnexal masses.

## Methods

### Study subjects

Between January 2010 and August 2012, 347 consecutive patients with clinically suspected adnexal disease underwent 3T MRI examinations before pelvic or laparoscopic surgery at our institution. The time interval between the MRI evaluation and surgery was less than one month (2–27 days; mean, 5 ± 12 days). Of these patients, 18 with histologically proven thecoma or fibrothecoma (42–81 years of age; average age, 59.9 ± 10.8 years) [2] were included in this study when we retrospectively retrieved the database on the Picture Archiving and Communication System (PACS). Other solid or predominantly solid adnexal masses, including histologically proven ligamentous leiomyomas (n = 14), primary ovarian masses (n = 20), and recurrent ovarian masses (n = 4), were included as the comparative group. Details of the samples studied are summarized in Table 1. Our institutional review board approved the study, and the requirement for the informed consent of all participants was waived. In this article, institutional review board was review board of Shanghai First People’s Hospital, Medical College, Shanghai Jiaotong University.

**Table 1:** The details of histopathological results in 56 solid adnexal lesions detected on 3T MRI

**Histology diagnosis**	**Numbers of lesions**
Thecoma and fibrothecoma	18
Leiomyoma	14
Other solid ovarian mass	24
Fibroma	2
Brenner tumor	3
Leiomyosarcoma	1
Clear cell adenocarcinoma	1
Granular cell tumor	2
Serous cystadenocarcinoma	5
Borderline cystadenoma	3
Dysgerminoma	1
Undefined adenocarcinoma	2
Recurrent solid ovarian cancer	4
Total	56

### Image acquisition

All MRI examinations were performed on a 3T system (Signa HD, General Electric Healthcare, Milwaukee, WI, USA) equipped with an eight-channel cardiac array coil. The scan range was from the umbilicus to the pubic symphysis in the caudocranial direction. For any larger lesion that could not be fully accommodated on axial imaging, a sagittal scanning sequence was performed to include as much of the entire lesion as possible. Routine MRI protocols were used for the assessment of the adnexal masses, which included axial fast spin-echo (FSE) T_1_-weighted images (T_1_WI), and sagittal FSE T_2_WI and fat-suppressed T2WI (FS T_2_WI) in the axial plane. A DWI-MRI sequence included an echo-planar imaging sequence with an array spatial-sensitivity-encoding technique (ASSET). The parameter details of the T_1_WI MR protocol were: repetition time (TR), 460 ms; echo time (TE), 10 ms; number of excitations (NEX), 2; and thickness, 6.0 mm. The parameter details of the T_2_WI MR protocol were: TR, 2400 ms; TE, 85 ms; NEX, 1; and thickness, 6.0 mm. The parameter details of the FS T_2_WI MR protocol were: TR, 3160 ms; TE, 90 ms; NEX, 2; and thickness, 6.0 mm. The parameter details of the DWI-MRI protocol were: TR, 3500 ms; TE, 61 ms; NEX, 6; and thickness, 6.0 mm; and the *b* value = 0 or 700 s/mm^2^. Liver acquisition with a volume acceleration (LAVA) sequence was used for contrast-enhanced pelvic imaging, and a power injector (Missouri Ulrich; Ulm, Germany) was used to inject the contrast material (Magnevist, Bayer Schering Pharma AG, Germany). The parameter details of the LAVA MR protocol were: TR, 3.4 ms; TE, 1.4 ms; NEX, 1; flip angle, 15°; band width, 125 kHz. The images were acquired in multiple phases of contrast medium enhancement in both the sagittal and axial planes (precontrast sagittal and axial oblique, and postcontrast at 20 s, 40 s, 60 s, and 80 s in the axial plane, and 120 s in the sagittal plane). Details of the parameters of image acquisitions are summarized in Table 2.

**Table 2:** Details of parameters for MRI imaging protocols

** Parameters**	**FSE-T**_**1**_**WI**	**FSE-T**_**2**_**WI**	**FS T2WI**	**EPI-DWI**	**LAVA**
Repetition/echo time (msec)	460/10	2400/85	3160/90	3500/61	3.4/1.4
NEX	2	1	2	6	1
Thickness(mm)	6	6	6	6	4
Field of view (mm)	36	36	36	40	38
Matrix	320 × 224	256 × 224	256 × 224	96 × 130	320 × 224
Flip angle (degrees)					15

### MRI image analysis

The MRI characteristics of each thecoma were recorded separately, including the following items: 1) lesion components (cystic, solid, cyst with septum, cyst with solid components, cyst with septum and solid components); 2) signal intensity on T_1_WI/T_2_WI and DWI-MRI was evaluated and recorded (hypo-, iso-, or hyperintensity on T_1_WI; hypo-, iso-, or hyperintensity on T_2_WI; low, intermediate, and high signal on DWI images). On T_1_WI, hypo-, iso-, and hyperintensity were similar for the pelvic fluid, pelvic wall muscle, and fat signal; on T_2_WI, hypo-, iso-, and hyperintensity were similar for the pelvic bone, pelvic wall muscle, and fat signal; on *b* = 700 mm^–2^/s DWI images, the low, intermediate, high signal intensities were similar for the pelvic bone, myometrium, and endometrium. After the intravenous injection of the contrast medium, the degree of lesion enhancement was graded as follows: 1, minor enhancement (clearly less than the myometrium); 2, mild enhancement (less than the myometrium); 3, moderate enhancement (similar to the myometrium); or 4, severe enhancement (more than the myometrium). Two observers (G.F.Z. and H.Z with 15 and 7 years of experience in gynecological imaging, respectively), who were blinded to the histological results, independently analyzed all the MRI datasets for each participant on a PACS terminal server. Consensus was achieved for any interobserver discrepancies in the evaluation of the adnexal lesions or G.F.Z.’s decision was arbitrarily accepted.

The ADCs were calculated by one observer (H.Z.) on a commercially available postprocessing workstation (GE Advantage Workstation 4.3, General Electric Healthcare, Milwaukee, WI, USA). Regions of interest were drawn manually in both the cystic and solid areas, with no more than three sites in each lesion on *b* = 700 mm^–2^/s DWI-MRI images. A circle or ellipsis with an area range of 160–320 mm^2^ was placed centrally in the targeted region. Only the lowest ADC value was used for the subsequent statistical analysis.

### Statistical analyses

Continuous variables were expressed as means ± standard deviation (SD) or as medians ± numerical ranges and compared with an unpaired *t* test if normally distributed (Mann–Whitney test if not normally distributed). SPSS version 13.0 (SPSS Inc., Chicago, USA) was used to perform all statistical analyses. A *p* value of less than 0.05 indicated a statistically significant difference.

## Results and discussion

The histological results revealed 18 ovarian thecomas in 18 patients (10 patients who underwent laparotomy and eight patients who underwent laparoscopic surgery). Two patients were perimenopausal and all the other patients were postmenopausal. All the thecomas were unilateral lesions, five of which were accompanied by other ovarian etiologies, including uterine fibroids (n = 3), endometrial polyps (n = 1), or fibroma and ligamentous leiomyoma (n = 1). All the patients presented to the gynecological department of our institution complaining primarily of a pelvic or abdominal mass, with no previous gynecological disease history, except in one patient with renal carcinoma (surgical resection five years earlier) incidentally detected on a routine follow-up abdominal MRI examination. There was no postmenopausal bleeding or vaginal discharge in this patient cohort. The baseline characteristics of the 18 thecomas are listed in Table 3.

**Table 3:** Baseline characteristics and ADC values of 56 patients with pathologically proven solid adnexal masses

** Histology diagnosis**	**Numbers**	**Age(years)**	**Maximum diameter(mm)**	**ADC(10-3/mm2/s)**
Thecoma	18	59.9 ± 10.8^*^	66.6 ± 46.0	1.20 ± 0.45
		59.5 (42–81)^$^	42.0 (23–80)	1.08 (0.70-2.20)
Leiomyoma	14	47.8 ± 16.5	53.2 ± 21.0	1.48 ± 0.42
		51.0 (24–73)	46.5 (27–106)	1.38 (0.90-2.50)
Other solid ovarian mass	24	54.6 ± 14.8	70.3 ± 42.1	1.26 ± 0.51
		58.0 (13–78)	66.5 (19–192)	1.09 (0.50-2.20)
Total	56	54.6 ± 14.6	64.8 ± 39.3	1.30 ± 0.47
		56.5 (13–81)	50.0 (19–192)	1.15 (0.50- 2.50)

^*^indicates mean ± standard deviation; ^$^ indicates median and range (minimum – maximum).

### MRI characteristics

**Figure 1:**
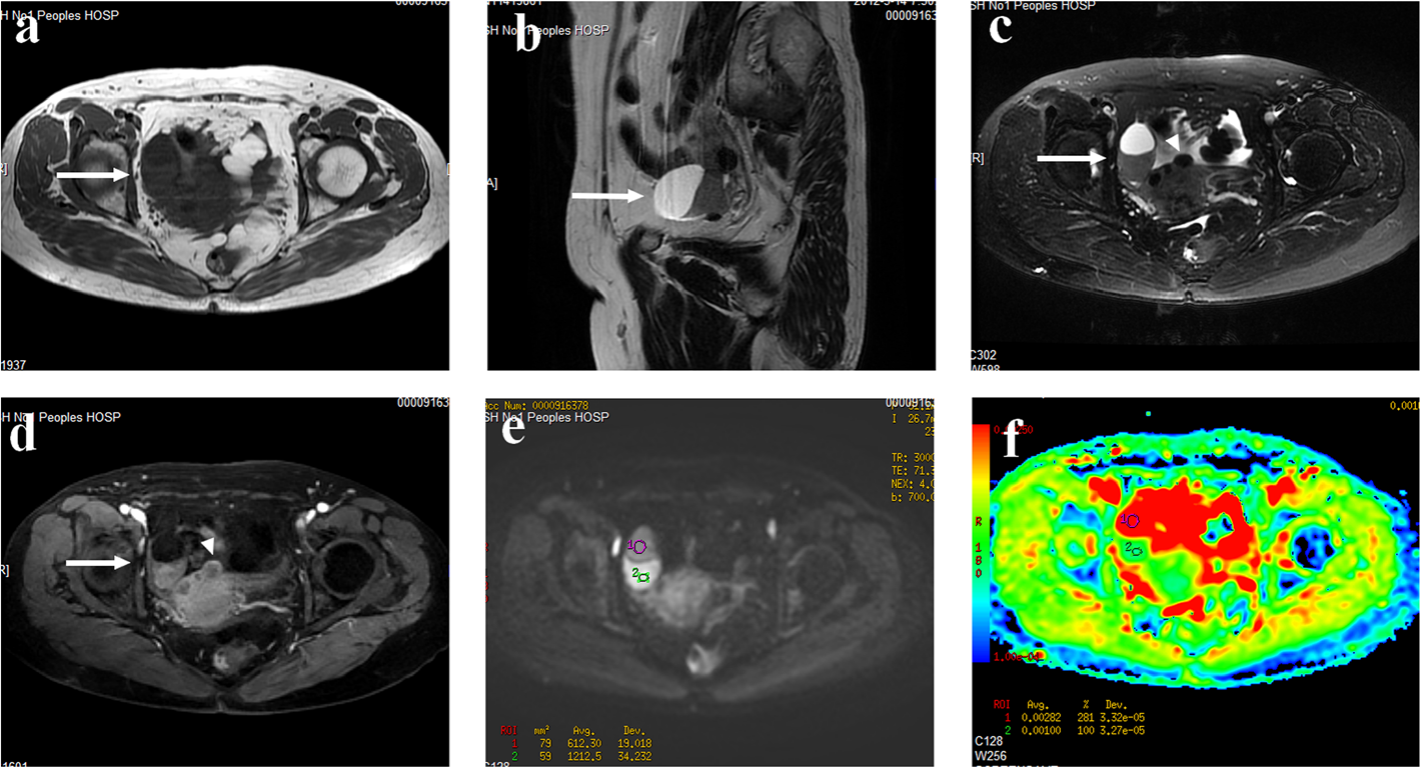
**A 60-year-old female patient with luteinized thecoma. (a)** Axial T_1_WI revealed a cystic solid mass with hypo–isointense signal in the right adnexal region (arrow). **(b)** On sagittal T_2_WI, the cystic component of the tumor gave a homogeneous hyperintense signal. Note that degenerated uterine fibroids (arrowhead) were also detected. **(c)** On fat-suppressed T_2_WI, the signal of the mass was similar to that in **(b)**. **(d)** On contrast-enhanced fat-suppressed T_1_WI, the solid components (arrow) of the lesion showed moderate enhancement, like that of the myometrium. **(e)** On DWI-MRI (*b* = 700 s/mm^2^), the upper part of lesion appears as a homogeneous isointense signal and the lower part as a hyperintense signal. **(f)** ADC map shows the marked hyperintense signal of the cystic components (T2 shine-through effect) and the isointense signal of the solid components. The ADC values at the corresponding sites are 2.82 × 10^–3^ mm^2^/s (cyst) and 1.00 × 10^–3^ mm^2^/s (solid).

**Figure 2:**
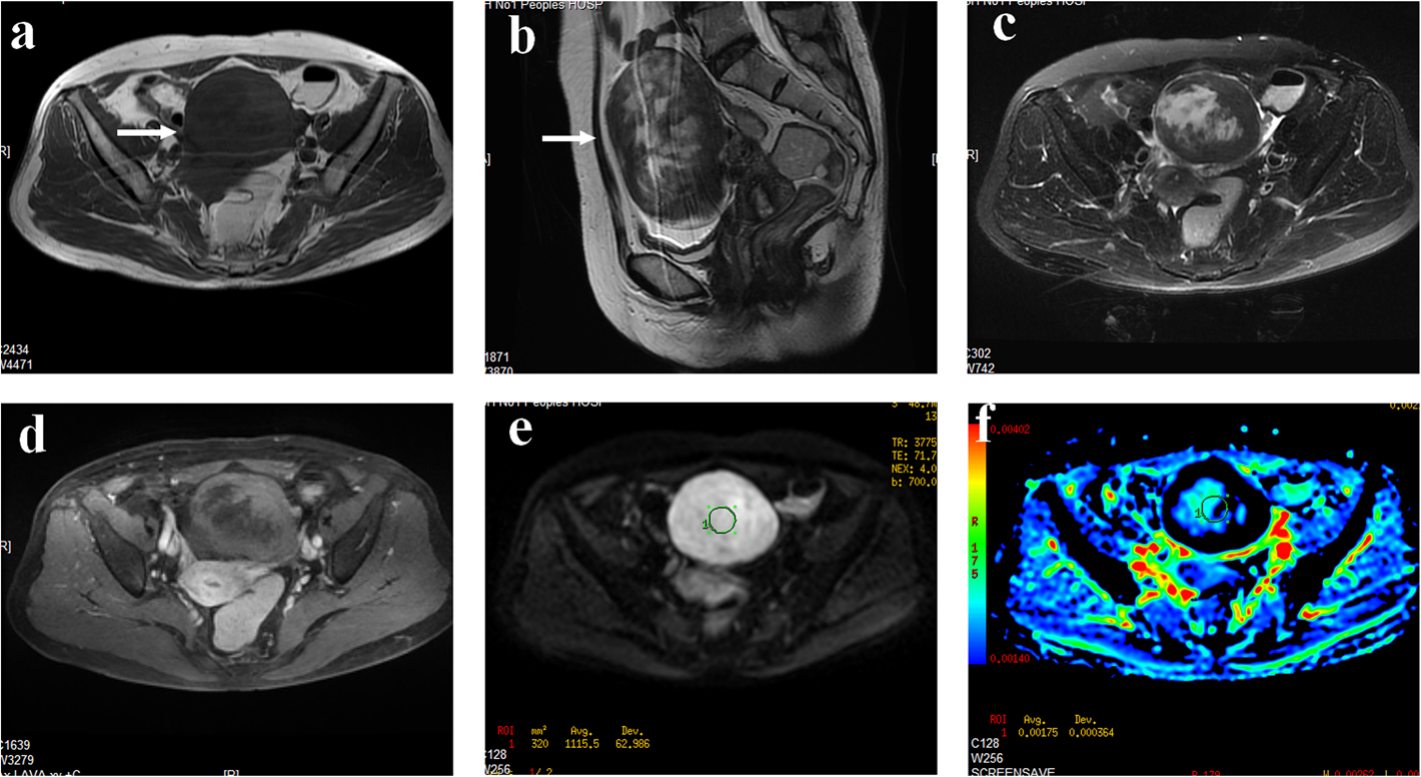
**A 57-year-old female patient with thecoma. (a)** Axial T_1_WI revealed a solid mass with a mostly iso–hypointense signal in the left adnexal region (arrow). **(b)** On sagittal T_2_WI, the solid tumor, 116 mm in maximum diameter, showed a heterogeneous signal with patchy high signal located eccentrically and an intermediate signal located peripherally. **(c)** On fat-suppressed T_2_WI, the signal of the mass was similar to that in **(b)**. **(d)** On contrast-enhanced fat-suppressed T_1_WI, the lesion showed mild enhancement, less than that of the myometrium. **(e)** On DWI-MRI (*b* = 700 s/mm^2^), the lesion appeared as a homogeneous hyperintense signal. **(f)** ADC map shows the hypointense signal with a marked peripheral hypointense signal. The ADC value at the corresponding site is 1.75 × 10^–3^ mm^2^/s.

In this patient population, all the thecomas were round or almost round solid masses with clear margins, except in one patient, who had cystic and solid components (pathologically proven to be luteinized thecoma) (Figure 1). In most cases, the lesion signals were homogeneously isointense on T_1_WI (17/18, 94.4%) and on T_2_WI (11/18, 61.1%). An isointense signal mixed with a patchy high signal (n = 4) (Figure 2) or low signal (n = 2) was also commonly observed on T_2_WI. No obvious pelvic fluid was seen in any patient. On the postcontrast images, nine lesions (50%) displayed minor enhancement, six mild enhancement, and three moderate enhancement. On *b* = 700 mm^–2^/s DWI images, 11 of the 18 lesions (11/18, 61.1%) displayed intermediate signal intensity, four high signal intensity, two low signal intensity, and one mixed signal intensity. The mean ADC value for the thecoma group was 1.20 ± 0.45 × 10^–3^ mm^2^/s. The details of the MRI characteristics of the ovarian thecomas are listed in Table 4.

**Table 4:** The details of baseline and MRI characteristics of 18 histologically proven thecoma and fibrothecoma in 18 patients

**Num**	**Age (years)**	**Maximum diameter (mm)**	**Components**	**MR signal (T**_**1**_**WI/T**_**2**_**WI/Enhancement)**	**DWI signal**	**ADC (10-3/mm2/s)**	**Accompanied lesions**
1	76	180.00	Solid	Iso/iso-hyperintensity/Mild	Low	2.23	
2	51	29.00	Solid	Iso/isointensity/Minor	High	0.82	Uterine fibroid
3	60	42.00	Cyst/solid	Iso/isointensity/Moderate	High	1.00	Fibroma, leiomyoma
4	57	116.00	Solid	Iso/iso-hyperintensity/Mild	Low/High	1.75	
5	57	89.00	Solid	Iso/isointensity/Minor	Intermediate	1.80	
6	65	23.00	Solid	Iso/isointensity/Minor	Intermediate	1.09	
7	81	64.00	Solid	Iso-hypo/isointensity/Mild	High	0.88	Endometrial polyps
8	50	32.00	Solid	Iso/isointensity/Mild	Intermediate	1.06	
9	59	37.00	Solid	Iso/iso-hypointensity/Minor	Intermediate	1.33	Uterine fibroid
10	55	42.00	Solid	Iso/isointensity/Minor	Intermediate	0.94	
11	43	135.00	Solid	Iso/iso-hyperintensity/Mild	Intermediate	1.10	
12	48	77.00	Solid	Iso/iso-hyperintensity/Moderate	High	1.14	Uterine fibroid
13	64	126.00	Solid	Iso/iso-hyperintensity/Mild	Intermediate	1.55	
14	71	77.00	Solid	Iso/isointensity/Mild	Intermediate	0.71	
15	42	26.00	Solid	Iso/isointensity/Minor	Intermediate	0.68	
16	69	34.00	Solid	Iso/isointensity/Minor	Intermediate	0.85	
17	67	42.00	Solid	Iso/isointensity/Minor	Intermediate	0.97	
18	64	25.00	Solid	Iso/hypointensity/Moderate	Low	1.47	

### Comparison with other solid ovarian masses

**Figure 3:**
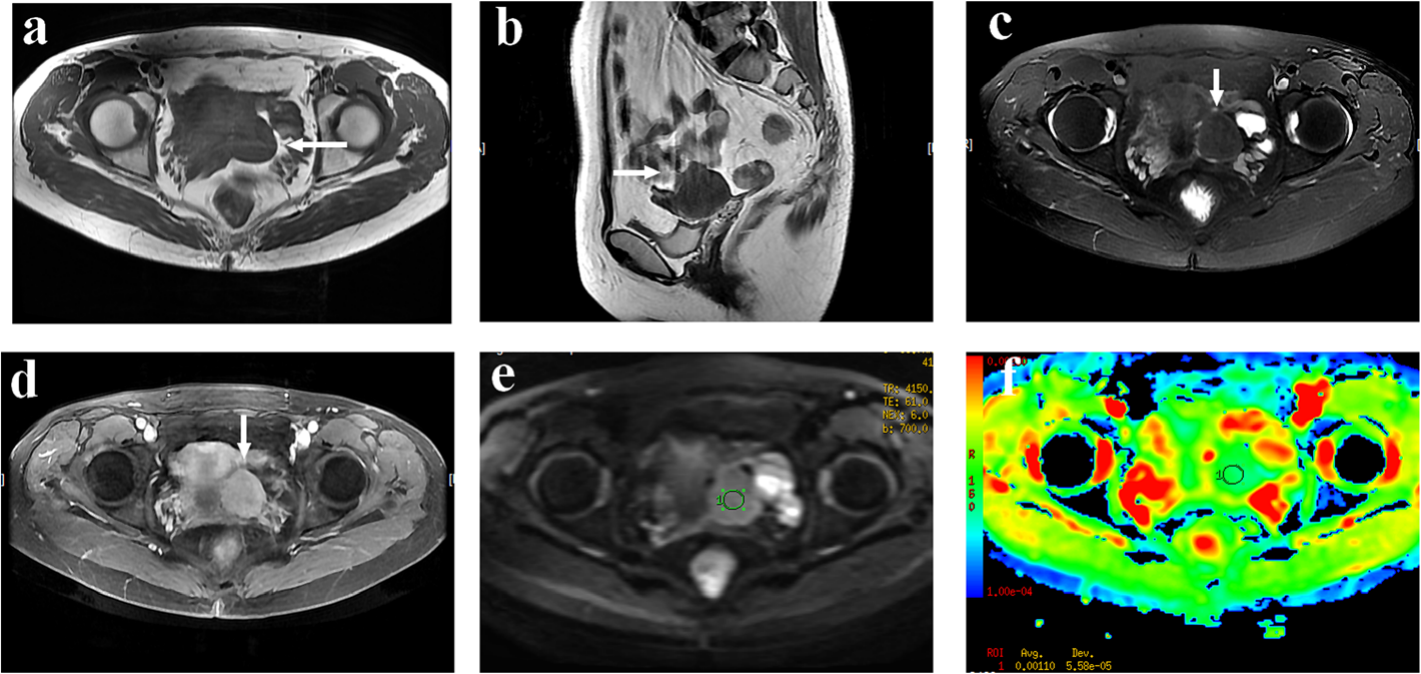
**A 55-year-old female patient with broad ligament fibroid.** A round solid mass with mostly isointense signal in the left adnexal region (arrow) was apparent on both T_1_WI **(a)** and T_2_WI **(b)**. **(c)** On fat-suppressed T_2_WI, the signal was similar to that in **(a)** and **(b)**. **(d)** On contrast-enhanced fat-suppressed T_1_WI, the lesion showed moderate enhancement, similar to that of the myometrium. **(e)** On DWI-MRI (*b* = 700 s/mm^2^), the lesion appeared as a homogeneous isointense signal. **(f)** The lesion shows intermediate signal on ADC map, with the ADC value of 1.10 × 10^–3^ mm^2^/s at the corresponding site.

**Figure 4:**
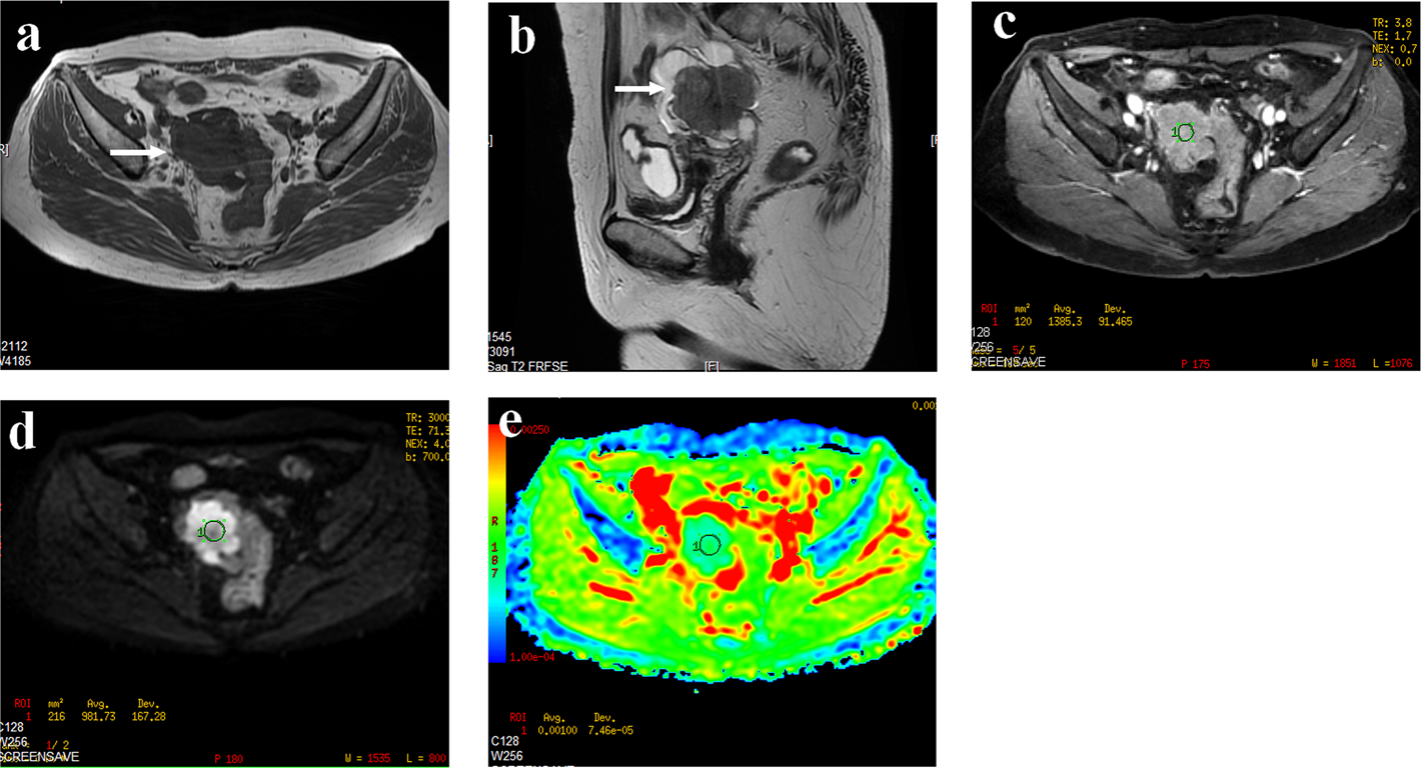
**A 59-year-old female patient with serous cystadenocarcinoma. (a)** Axial T_1_WI revealed a lobulated solid mass with isointensity in the right adnexal region (arrow). **(b)** On sagittal T2WI, the solid tumor displayed homogeneous isointensity. **(c)** On contrast-enhanced fat-suppressed T_1_WI, the lesion showed mild enhancement. **(d)** On DWI-MRI (*b* = 700 s/mm^2^), the lesion appeared as hyperintensity. **(e)** ADC map demonstrates homogeneous isointensity, with an ADC value of 1.00 × 10^–3^ mm^2^/s.

**Figure 5:**
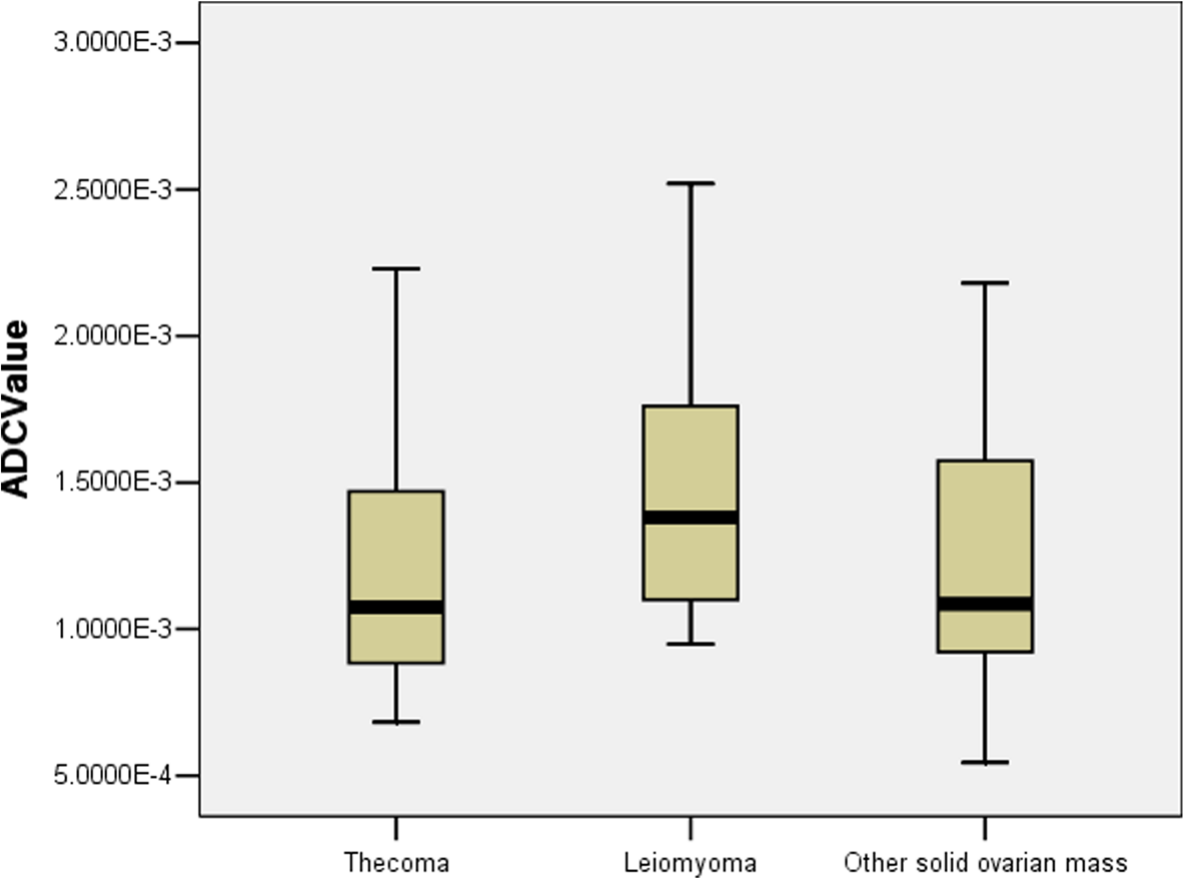
**Stem-and-leaf plots of the calculated ADC values (× 10**^**–3 **^**mm**^**2**^**/s) for the three groups.** The median ADC value (1.08) in the thecoma group was almost equal to that of the other solid ovarian masses (1.09), but less than that of the leiomyoma group (1.38). There was no significant difference between the groups.

**Figure 6:**
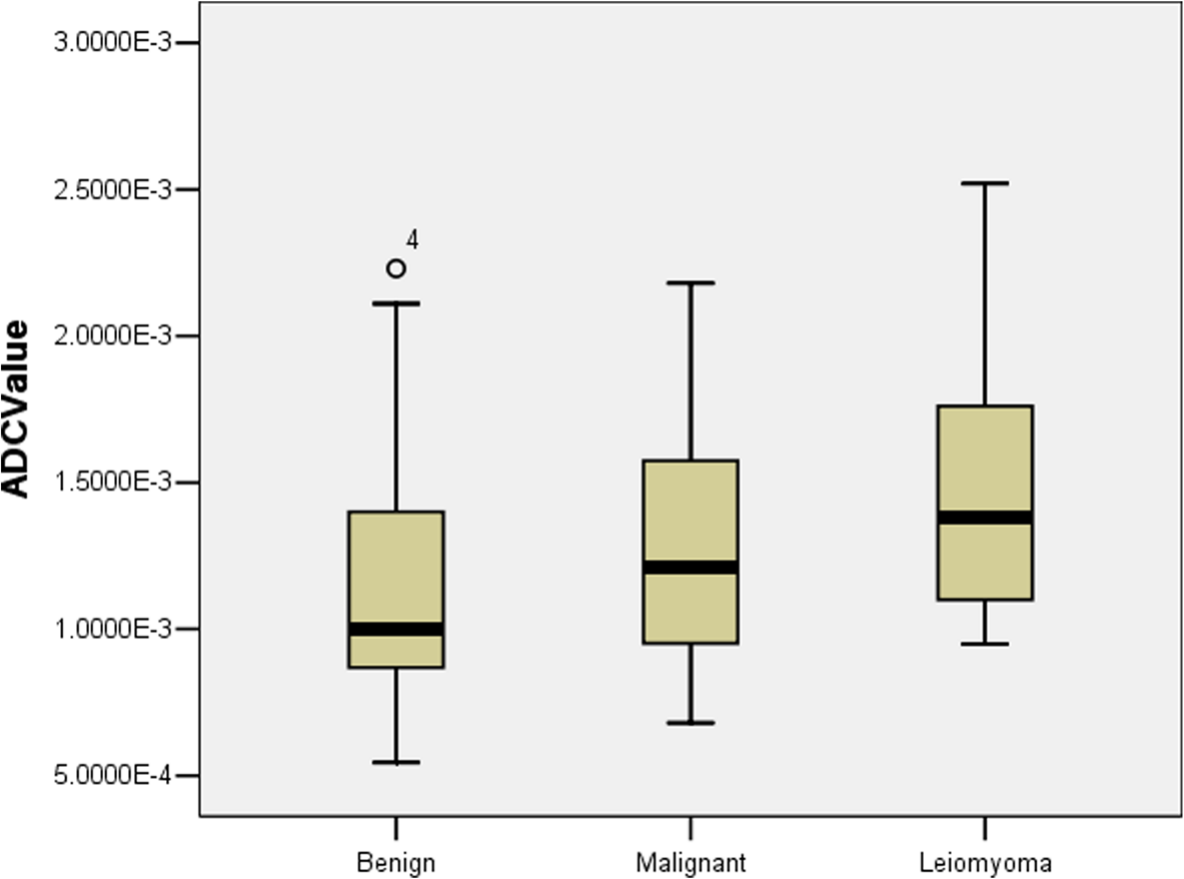
**Stem-and-leaf plots of the calculated ADC values (10**^**–3**^ **× mm**^**2**^**/s) of the three groups.** The median ADC (1.00) in the benign solid group was less than that in the malignant solid group (1.21) or the leiomyoma group (1.38). The difference between the benign solid group and malignant solid group was not significant (*p* = 0.234), whereas the difference between the benign solid group and the leiomyoma group was significant (*p* = 0.043).

In this study, 14 ligamentous leiomyomas (Figure 3) and 28 solid ovarian masses, including 20 primary ovarian tumors (Figure 4) and four recurrent solid ovarian cancers, were included in the comparative group (Table 1). The baseline characteristics of the 42 other solid ovarian masses are summarized in Table 5. The mean age of the thecoma group (59.9 ± 10.8 years) was clearly higher than that of the leiomyoma group (47.8 ± 16.5 years; *p* = 0.018), but similar to that of patients with other solid ovarian masses (54.6 ± 14.8 years; *p* = 0.201; Table 6). There was no significant difference in the maximum lesion diameters of each group, although the maximum diameter in other solid ovarian mass group was a little larger than the diameters of the other groups. The mean ADC value of the thecoma group (1.20 ± 0.45 × 10^–3^ mm^2^/s) was almost equal to that of the group of other solid ovarian masses (1.26 ± 0.51 × 10^–3^ mm^2^/s; Figure 5), but lower than the mean ADC for leiomyomas (1.48 ± 0.42 × 10^–3^ mm^2^/s), although not significantly so (*p* = 0.086). When the ovarian solid masses were categorized into a benign group (including 18 thecomas, two fibromas, and three Brenner tumors) and a malignant group (19 lesions), there was no significant difference in their maximum diameters (62.3 ± 42.2 and 76.4 ± 44.5, respectively) or ADC values (1.16 ± 0.47 × 10^–3^ and 1.33 ± 0.48 × 10^–3^ mm^2^/s, respectively), but age differed significantly (60.7 ± 10.2 and 52.3 ± 15.4 years, respectively; *p* = 0.042; Table 6). The ADCs of the benign group and leiomyoma group differed significantly (*p* = 0.043), although a wide overlap between the two groups was observed (Figure 6).

**Table 5:** Baseline characteristics and ADC values (mean ± SD) of 42 patients with pathologically proven other solid ovarian masses

**Pathology group**	**Numbers**	**Age(years)**	**Maximum diameter(mm)**	**ADC(10-3/mm2/s)**
Benign	23	60.7 ± 10.2^*^	62.3 ± 42.2	1.16 ± 0.47
		60.0 (42–81)^$^	42.0 (19–180)	1.00 (0.50-2.20)
Malignant	19	52.3 ± 15.4	76.4 ± 44.5	1.33 ± 0.48
		52.0 (13–78)	70.0 (20–192)	1.21 (0.70-2.20)
Total	42	56.9 ± 13.3	68.7 ± 43.3	1.24 ± 0.48
		59.0 (13–81)	61.0 (19–192)	1.08 (0.50-2.20)

^*^indicates mean ± standard deviation; ^$^indicates median and range (minimum – maximum).

**Table 6:** **The statistically significant difference (*p*value) of baseline characteristics and ADC values within three groups in 56 patients with pathologically proven solid adnexal masses**

** Pathology group**	**Age**	**Max diameter**	**ADC(10-3/mm2/s)**
Thecoma vs Leiomyoma	0.018	0.322	0.086
Leiomyoma vs Other solid mass	0.198	0.167	0.187
Thecoma vs Other solid mass	0.201	0.791	0.693
Benign vs Malignant	0.042	0.299	0.234
Leiomyoma vs Benign	0.006	0.459	0.043
Leiomyoma vs Malignant	0.424	0.081	0.378

Ovarian thecoma is a solid ovarian tumor of gonadal stromal-cell origin, although cystic degeneration and varying degrees of edema can be observed in larger lesions [3-5]. Generally, ovarian thecoma is considered a benign disease, although some anecdotal malignant cases have been reported [2,3]. It is sometimes difficult to differentiate these solid tumors from broad ligament leiomyomas because their imaging features on ultrasound are similar [12]. Ascites and pleural effusions accompanying the tumor (Meigs syndrome) may be present in some cases [13], prompting an easy misdiagnosis of ovarian cancer. From this perspective, radiologists must be thoroughly familiar with the imaging characteristics of these tumors to ensure their capacity to make a correct preoperative diagnosis. When we searched the literature, we found that studies focusing on the MRI features of thecomas or fibrothecomas were alarmingly limited, although MRI has been widely used in the clinical context for any ovarian masses that are indeterminate on computed tomography or US. In this study, we analyzed 18 cases of histologically proven thecoma and fibrothecoma with conventional MRI and DWI. To our knowledge, no study of ovarian thecoma in a large cohort sample with 3T MRI has previously been published.

In this study, the mean age at onset of thecoma was 59.9 years and 16 patients (88.9%) were in the postmenopausal period. Our results are consistent with the literature in that thecoma predominantly affects postmenopausal women, with a mean age of 59 years [2,3]. The peak incidence occurred significantly later in the thecoma group than in the intraligamentous leiomyoma group (47.8 years; *p* = 0.018). In this study, the lesions most often accompanying thecoma were uterine fibroids or intraligamentous leiomyoma (4/18). In the 19 cases studied by Li XC et al., endometrial abnormalities (two cases with hyperplasia and one case with endometrial carcinoma) accompanying the thecoma were described as resulting from elevated levels of estrogen [3]. In contrast to other studies [3,4,14], we observed no ascites or pleural effusions in this study. The mechanisms underlying these phenomena still require clarification, but they may be related to the hormonal activity of the thecoma.

According to the MRI findings in the present study, all the thecomas were solid-component masses, regardless of the lesion size, except for one cystic and solid mass, with a pathological diagnosis of luteinized thecoma, which is described in the literature as a rare condition [15]. In another study of 19 patient samples, the authors reported four lesions that presented as mostly cystic masses on cross-sectional imaging [3], which could represent cystic degeneration or stromal edema. In these cases, it is often impossible to distinguish these tumors from ovarian epithelial tumors. Most of the thecomas examined in our study appeared homogeneously isointense either on T_1_WI (17/18, 94.4%) and T_2_WI (11/18, 61.1%) or on DWI-MRI (11/18, 61.1%). However, we did find that the larger lesions (four lesions > 100 mm in maximum diameter) displayed a more heterogeneous signal on T_2_WI (appearing as an iso–hyperintense signal) than did the smaller tumors. Our observations are basically consistent with those of the studies in the literature [3-5]. In a study of 12 fibromas and fibrothecomas, Troiano et al. reported that the patchy high signal on the T_2_WI images (representing cystic degeneration) occurred centrally or eccentrically, whereas the low signal (representing many fibro or thecal cellular components) was more often peripheral [5]. We also noted this characteristic in the two largest tumors (Figure 2). Because this feature is never present in ovarian epithelial tumors, it may be an important MRI clue to the etiology of the mass if this is unclear from well-documented morphological criteria. In terms of lesion enhancement, Shinagare et al. studied the MRI characteristics of 25 ovarian fibromas and 10 fibrothecomas, and demonstrated that the average maximum percentage enhancement of the fibromas and fibrothecomas was significantly lower than the enhancement of the myometrium and fibroids [4]. Of the 18 tumors examined in the present study, 15 (83.3%) showed weak enhancement relative to that of the myometrium. Thus, our results corroborate the view that most thecomas show minor enhancement, which may be useful in discriminating them from broad ligament fibroids.

DW-MRI is a functional imaging technique that provides information about water mobility (Brownian movement), tissue cellularity, and the integrity of cellular membranes, and is gradually becoming part of the standard imaging protocols used to evaluate obstetric and gynecological diseases [16]. The combination of conventional MRI and DWI-MRI could improve lesion detection and the better categorization of adnexal lesions [17], and with the advantages of a contrast-free technique and a shorter acquisition time (1–2 min), the use of the DWI-MRI sequence can easily be added to routine imaging protocols. To the best of our knowledge, no DWI-MRI evaluation of thecomas in relatively larger numbers of patients and their comparison with adnexal leiomyomas has been reported until now. In the present study, most thecomas (11/18, 61.1%) displayed intermediate signals (similar to that of the myometrium) on DWI-MRI, which may be attributable to the presence of many fibroblasts and thecal cells [2]. Because thecomas are benign in nature, their DWI-MRI characteristics may differ from those of malignant ovarian tumors. In a study of 140 primary ovarian lesions, the authors reported that most of the malignant tumors (27/42) produced a high signal on DWI-MRI [11]. In the present study, the mean ADC for the thecoma group was lower than that of the leiomyoma group or that of the other solid ovarian masses, although none of these differences were significant. In contrast to our study, Bakir et al. reported that the mean ADC for fibrothecoma was higher than that for ordinary leiomyoma [18]. The assessment of only two fibrothecomas in that study may be responsible for this discrepancy. The utility of ADC in the categorization adnexal solid lesions has been reported in only a few studies [11,18,19], so the conclusions still are contentious because different *b* values were selected, varying pathologies were examined, and the samples studied had different volumes. In this study, the ADCs for the solid ovarian masses in the benign group (23 lesions) were slightly lower than those of the malignant group (19 lesions), but not significantly so. The benign group, which included thecomas, fibromas, and Brenner tumors, all showed many closely arrayed spindle fibroids and thecal cells, which would obviously reduce the ADCs on DWI images. Our results are consistent with those of other recently reported studies [11,18]. It is noteworthy that there was a significant difference in the ADCs for the benign solid ovarian tumors and leiomyomas (*p* = 0.043). This result confirms that ADCs can be used to differentiate benign ovarian solid tumors from ligamentous leiomyomas.

There were some limitations to this study. First, there was an inherent selection bias because the study was retrospective. However, all the patients underwent prospective MRI examinations, which may have partly offset this bias. The limited study sample size could also have influenced the final results. Second, we compared our results using 3T MRI with other studies based on 1.5T MRI. The potential influence of this difference in magnetic field may have made our comparison inaccurate. Third, the ADCs were manually measured on the regions of interest based on individual habits. The lack of standardization in calculating ADC may also have influenced the final results.

## Conclusions

Ovarian thecoma or fibrothecoma often manifests as a solid mass with homogeneous isointensity on both T_1_WI/T_2_WI and DWI-MRI. There was no significant difference in the ADCs for thecoma and other adnexal solid masses, although the ADCs of the thecomas and fibrothecomas were lower than those of the leiomyomas. A significant difference in the ADCs for benign solid ovarian masses and ligamentous leiomyomas was observed, but this parameter was not useful in differentiating benign from malignant solid ovarian tumors.

## Competing interests

The authors declare that they have no competing interest.

## Authors’ contributions

Guarantor of integrity of entire study, GFZ.; study concepts/study design or data acquisition or data analysis/interpretation, all authors; manuscript drafting or manuscript revision for important intellectual content, GFZ, HZ; approval of final version of submitted manuscript, all authors; literature research, HZ; clinical studies, GFZ, HZ, TPW; statistical analysis, HZ; and manuscript editing, HZ. All authors read and approved the final manuscript.

## References

[B1] Chen VW Ruiz B Killeen JL TimothyR C Wu XC Catherine NC Holly LH Pathology and classification of ovarian tumors Cancer 2003 97 2631 2642 10.1002/cncr.11345 12733128

[B2] Nocito AL Sarancone S Bacchi C Tellez T Ovarian thecoma: Clinicopathological analysis of 50 cases Ann Diagn Pathol 2008 12 12 16 10.1016/j.anndiagpath.2007.01.011 18164409

[B3] Li X Zhang W Zhu G Sun C Liu Q Shen Y Imaging features and pathologic characteristics of ovarian thecoma J Comput Assist Tomogr 2012 36 46 53 10.1097/RCT.0b013e31823f6186 22261769

[B4] Shinagare AB Meylaerts LJ Laury AR Mortele KJ Mri features of ovarian fibroma and fibrothecoma with histopathologic correlation Am J Roentgenol 2012 198 W296 W303 10.2214/AJR.11.7221 22358029

[B5] Troiano RN Lazzarini KM Scoutt LM Lange RC Flynn SD McCarthy S Fibroma and fibrothecoma of the ovary: Mr imaging findings Radiology 1997 204 795 798 9280262 10.1148/radiology.204.3.9280262

[B6] Nakayama T Yoshimitsu K Irie H Hitoshi A Tsuyoshi T Akihiro N Asayama Y Yoshiki A Kunishige M Daisuke K Shuji M Hitoo N Hiroshi H Diffusion-weighted echo-planar mr imaging and adc mapping in the differential diagnosis of ovarian cystic masses: Usefulness of detecting keratinoid substances in mature cystic teratomas J Magn Reson Imaging 2005 22 271 278 10.1002/jmri.20369 16028258

[B7] Adusumilli S Hussain HK Caoili EM William JW John PM Timothy DJ Chen Q Desjardins B Mri of sonographically indeterminate adnexal masses Am J Roentgenol 2006 187 732 740 10.2214/AJR.05.0905 16928938

[B8] Bazot M Nassar-Slaba J Thomassin-Naggara I Cortez A Uzan S Darai E Mr imaging compared with intraoperative frozen-section examination for the diagnosis of adnexal tumors; correlation with final histology Eur Radiol 2006 16 2687 2699 10.1007/s00330-006-0163-z 16547708

[B9] Sohaib SA Mills TD Sahdev A Webb JAW VanTrappen PO Jacobs IJ Reznek RH The role of magnetic resonance imaging and ultrasound in patients with adnexal masses Clin Radiol 2005 60 340 348 10.1016/j.crad.2004.09.007 15710137

[B10] Thomassin-Naggara I Toussaint I Perrot N Rouzier R Cuenod CA Bazot M Darai E Characterization of complex adnexal masses: Value of adding perfusion- and diffusion-weighted mr imaging to conventional mr imaging Radiology 2011 258 793 803 10.1148/radiol.10100751 21193596

[B11] Zhang H Zhang G-F He Z-Y Li Z-Y Zhu M Zhang G-X Evaluation of primary adnexal masses by 3t mri: Categorization with conventional mr imaging and diffusion-weighted imaging J Ovarian Res 2012 5 33 10.1186/1757-2215-5-33 23148860 PMC3576319

[B12] Murase E Siegelman ES Outwater EK Perez-Jaffe LA Tureck RW Uterine leiomyomas: Histopathologic features, mr imaging findings, differential diagnosis, and treatment Radiographics 1999 19 1179 1197 10489175 10.1148/radiographics.19.5.g99se131179

[B13] Tanaka YO Tsunoda H Kitagawa Y Ueno T Yoshikawa H Saida Y Functioning ovarian tumors: Direct and indirect findings at mr imaging1 Radiographics 2004 24 S147 S166 10.1148/rg.24si045501 15486238

[B14] Liu H Hao S Li W Giant malignant ovarian fibrothecoma involved with retroperitoneal structures mimicking a retroperitoneal sarcoma Arch Gynecol Obstet 2009 279 763 765 10.1007/s00404-008-0799-9 18813938

[B15] Athula Kaluarachchi JPM Batcha TM Preethika A Luteinized ovarian thecoma in a postmenopausal women presenting with virilization Obstet Gynecol Int 2009 492386 10.1155/2009/492386 19946643 PMC2778823

[B16] Sala E Rockall A Rangarajan D Kubik-Huch RA The role of dynamic contrast-enhanced and diffusion weighted magnetic resonance imaging in the female pelvis Eur J Radiol 2010 76 367 385 10.1016/j.ejrad.2010.01.026 20810230

[B17] Punwani S Diffusion weighted imaging of female pelvic cancers: concepts and clinical applications Eur J Radiol 2011 78 21 29 10.1016/j.ejrad.2010.07.028 20801592

[B18] Bakir B Bakan S Tunaci M Bakir VL Iyibozkurt AC Berkman S Bengisu E Salmaslioğlu A Diffusion-weighted imaging of solid or predominantly solid gynaecological adnexial masses: Is it useful in the differential diagnosis? Br J Radiol 2011 84 600 611 10.1259/bjr/90706205 21081581 PMC3473502

[B19] Takeuchi M Matsuzaki K Nishitani H Diffusion-weighted magnetic resonance imaging of ovarian tumors: differentiation of benign and malignant solid components of ovarian masses J Comput Assist Tomogr 2010 34 173 176 10.1097/RCT.0b013e3181c2f0a2 20351498

